# Mechanical Stress Inhibits Early Stages of Endogenous Cell Migration: A Pilot Study in an Ex Vivo Osteochondral Model

**DOI:** 10.3390/polym12081754

**Published:** 2020-08-06

**Authors:** Maria L. Vainieri, Mauro Alini, Avner Yayon, Gerjo J. V. M. van Osch, Sibylle Grad

**Affiliations:** 1 AO Research Institute Davos, 7270 Davos, Switzerland; letizia.vainieri@aofoundation.org (M.L.V.); mauro.alini@aofoundation.org (M.A.); 2 Department of Orthopaedics, Erasmus MC, University Medical Center Rotterdam, 3015 CN Rotterdam, The Netherlands; g.vanosch@erasmusmc.nl; 3 ProCore Ltd., Weizmann Science Park, 7 Golda Meir St., Ness Ziona 70400, Israel; yayon@procore-bio.com; 4 Department of Otorhinolaryngology, Erasmus MC, University Medical Center Rotterdam, 3015 CN Rotterdam, The Netherlands; 5 Department of Biomedical Engineering, University of Technology Delft, 2628 CD Delft, The Netherlands; 6 Department of Health Sciences and Technology, ETH Zurich, 8092 Zurich, Switzerland

**Keywords:** biomaterial, hydrogel, cartilage, osteochondral, mechanical loading, endogenous cell recruitment

## Abstract

Cell migration has a central role in osteochondral defect repair initiation and biomaterial-mediated regeneration. New advancements to reestablish tissue function include biomaterials and factors promoting cell recruitment, differentiation and tissue integration, but little is known about responses to mechanical stimuli. In the present pilot study, we tested the influence of extrinsic forces in combination with biomaterials releasing chemoattractant signals on cell migration. We used an ex vivo mechanically stimulated osteochondral defect explant filled with fibrin/hyaluronan hydrogel, in presence or absence of platelet-derived growth factor-BB or stromal cell-derived factor 1, to assess endogenous cell recruitment into the wound site. Periodic mechanical stress at early time point negatively influenced cell infiltration compared to unloaded samples, and the implementation of chemokines to increase cell migration was not efficient to overcome this negative effect. The gene expression at 15 days of culture indicated a marked downregulation of matrix metalloproteinase *(MMP)13* and *MMP3,* a decrease of *β1* integrin and increased mRNA levels of actin in osteochondral samples exposed to complex load. This work using an ex vivo osteochondral mechanically stimulated advanced platform demonstrated that recurrent mechanical stress at early time points impeded cell migration into the hydrogel, providing a unique opportunity to improve our understanding on management of joint injury.

## 1. Introduction

Articular cartilage plays a key role in the function of joints, and when damaged it becomes inefficient to withstand harsh conditions over time, posing a significant challenge among clinicians. The very poor intrinsic healing capacity of this tissue in combination with the high incidence of trauma place at risk many asymptomatic young and healthy patients toward the evolution of degenerative conditions with reduced possibility of interventions [1]. 

Surgical procedures including microfracture and osteochondral allografts are being applied in clinical practice. While the former is far from being successful in replacing the damaged cartilage by repair tissue with long-lasting hyaline properties [2], the latter is often a last resource revision surgery after failed attempts of cartilage reconstruction [3], in order to address the subchondral changes seen in the revision setting. The invasiveness of this procedure due to the removal of a healthy cartilage portion together with the potential graft-size mismatch, may hamper the efficacy of this intervention. Emerging opportunities with cell-based repair approaches are considered, such as autologous chondrocytes implantation (ACI) [4], matrix-assisted ACI (MACI) [5] and transplantation of autologous mesenchymal stem cells (MSCs) [6]. Studies comparing patients treated with these strategies have shown similar improvements in term of clinical outcome, although longer periods of randomized trials are required to conclude effective regeneration [7,8]. Nevertheless, cell therapy faces limitations in clinics in term of costs, safety and quality controls [9]. With the perspective to circumvent these issues and take advantage of bone marrow and bone lining stem/progenitor cells, biomaterial implantation is used to enhance the natural healing process that microfracture affords. An example of this procedure called autologous matrix-induced chondrogenesis (AMIC) is using a collagen membrane to enhance cartilage repair by endogenous progenitor cells [10]. While no difference was found in the outcome between ACI and AMIC for treating cartilage defects in a two-year follow-up [11], this technique promoted cell-free alternatives via the conception of an instructed microenvironment toward regeneration.

In recent years the modulation of biochemical and biophysical cues, when considering the design of biomaterials used as 3D templates for tissue regeneration, has advanced our understanding of cartilage repair processes [12]. These determinants control both extracellular matrix environment and cell behavior such as cell adhesion, migration and differentiation, which are key processes for successful formation of functional tissues. Indeed, a number of studies have demonstrated that incorporation of small oligopeptides (such as RGD, [13,14]) conjugated to the backbone of polymers can improve their function as adhesive materials; the presence of chemotactic stimuli in hydrogels, such as platelet-derived growth factor-BB (PDGF-BB) or stromal cell-derived factor 1 (SDF-1α) [15,16], can enhance cellular migration. Notably, the modulation of matrix-metalloproteinase (MMP) activity combined with RGD peptides or the addition of micro-RNAs were shown to be able to promote endogenous cell recruited cartilage repair [17,18], while the introduction of MMPs can also enhance graft integration to the wound site [19].

In agreement with a body of evidence from literature, our previous work on cartilage healing using in vitro studies and a model for osteochondral defect repair after subcutaneous implantation in mice suggest that stiffer materials represent a barrier to endogenous healing [16], where matrix limits infiltration and remodeling near injury sites. Cell migration has a critical role in the early process of biomaterial-assisted tissue repair. While several factors are important for cartilage repair success, to render cell-free technologies clinically feasible, mechanical factors should be considered to evaluate their performance in a physiological joint environment. Mechanical loading plays an important role for spontaneous and biomaterial guided chondral and osteochondral defect repair. Several in vitro studies have demonstrated that compressive and/or shear load promoted the anabolic phenotype, cartilaginous matrix synthesis of articular chondrocytes and chondrogenic differentiation in mesenchymal stem cells [20,21,22,23,24]; these findings led to the definition of regenerative rehabilitation principles in translational orthopedics [25]. Gene expression is affected by mechano-transduction, which results in rapid and long-term cellular changes mediated by integrin-dependent Ras homolog family member A (RhoA) signaling and downstream actin dynamics [26]. Mechanical compression of glycoprotein-polysaccharide complexes, present at the cell surface to exert electrosteric repulsion to the extracellular matrix (ECM) around integrin receptors, promotes integrin activation and clustering in a kinetic trap manner. This process facilitates focal adhesion to the matrix and contraction, which results in different cell responses depending on ECM stiffness, cell distribution and density to control proliferation and differentiation [27,28]. Matrix is actively organized by cells through their integrins, with the actomyosin machinery allowing them to pull or push on collagen fibers to then establish a new mechanical state [29]. In condition of high tension, tenascin transcription increases and reduces cellular interaction by decreasing Rho activity and contraction ability, suggesting a key role of this protein in the negative feedback loop to promote mechanical homeostasis under high stress condition [30,31]. These observations suggest that an appropriate loading regime may facilitate the development of a stable cartilage phenotype. In an in vivo rabbit osteochondral defect model treated with cell-free porous poly(lactic-co-glycolic acid) graft implants, daily treadmill exercise resulted in improved outcome in terms of hyaline cartilage tissue formation [32]. However, the effect of early mechanical stimulation on the recruitment of endogenous cells for cartilage and osteochondral defect repair remains largely unknown [33].

The success of material-based systems for osteochondral defect repair depends on the ability of the scaffolds to sustain compressive, shear and tensile forces during joint loading. Although most hydrogels are not ideal materials to resist complex motion, mechanical properties can be enhanced by modifying polymers with functional groups to form hydrophilic structures and increase the crosslinking density in the network [34]. Fibrin/hyaluronan (FB/HA) hydrogel formulation had previously been investigated in vitro and in vivo as suitable material for cell infiltration for repair of articular cartilage defects; also, the hydrogel has been shown to withstand mechanical loading [16,17,35]. With such regenerative tools in our hands, our goal was to test the influence of applied extrinsic forces on the endogenous cell recruitment process by using our custom-made joint bioreactor. Towards this aim, we used an ex vivo mechanically stimulated osteochondral defect explant model filled with FB/HA hydrogel in the presence or absence of PDGF-BB or SDF-1α to further enhance cell infiltration. In the present pilot study, we addressed the hypothesis that mechanical compression and shear would modulate the early stage of cell migration into a FB/HA hydrogel implanted in an osteochondral defect ex vivo.

## 2. Materials and Methods

### 2.1. Osteochondral Tissue Harvest and Culture

Osteochondral explants were harvested from stifle joints of five to eight-months-old calves, obtained from a local abattoir (Metzgerei Angst AG, Zurich, Switzerland) within 48 h of slaughter. Previous studies using the same timeframe have shown explant viability preservation for up to 28 days [36,37]. Cylindrical osteochondral plugs were obtained as previously described [36] with an 8 mm diameter custom-made coated trephine drill (Peertools AG, Ftan, Switzerland). The subchondral bone part was trimmed to obtain a final explant height of 6 mm. To generate osteochondral defects of 3 mm depth, a 4 mm diameter trephine drill was used (Brutsch-Ruegger, Urdorf, Switzerland). Subsequently osteochondral explants were placed in bioreactor holders containing 2% low-gelling agarose (SeaPlaque Agarose, Lonza, Rockland, USA), to cover the bone part and prevent cell outgrowth from the subchondral bone. Then, explants were cultured in Dulbecco’s modified Eagle medium (DMEM-HG, 4.5 g/L-glucose; Gibco, Dublin, Ireland) supplemented with 1% insulin-transferrin-selenium (ITS, Corning, New York, NY, USA), non-essential amino acids, 1% penicillin-streptomycin (Gibco), 25 μg/mL ascorbic acid-2-phosphate (AA-2-P, Sigma-Aldrich, Saint Louis, MO, USA), amino caproic acid (Sigma-Aldrich) and 100 nM dexamethasone (Sigma-Aldrich) at 37 °C and 5% CO_2_. The medium, referred to as chondro-permissive medium, was changed three times per week.

### 2.2. Fibrin-HA Hydrogel Preparation and Incorporation of PDGF-BB or SDF1α

FB/HA conjugates were synthesized via a two-step reaction as previously described [38]. Final concentrations of 6.25 mg/mL FB and 1.96 mg/mL of HA-active ester solution (FB/HA *w/v* ratio of 3.2:1) were used with HA molecular weight of 235 kDa (LifeCore Biomedical, LLC, Chaska, MN, USA). Briefly, HA was first reacted with a mixture of 1-ethyl-3-(3-dimethylaminopropyl) carbodiimide (EDC; Sigma, Rehovot, Israel) and *N*-hydroxysuccinimide (NHS; Sigma, Rehovot, Israel) to convert part of its carboxylic groups to NHS-active ester moieties. In a second step, a buffered solution of fibrinogen (Omrix, Ness Ziona, Israel) was reacted with the HA active ester solution to produce a clear FB/HA conjugate solution. Hydrogels were then prepared by mixing thrombin solution (50 U/mL, Sigma-Aldrich) containing calcium chloride (1M CaCl_2_) with FB/HA conjugate and polymerizing at 37 °C for 30 min. The rheological features of the resulting hydrogels were characterized in previous works [16,38].

PDGF-BB or SDF-1α (both Peprotech, London, UK) were added to the FB/HA conjugate solution prior polymerization to obtain final concentrations of 2 µg/mL of PDGF-BB or 10 µg/mL of SDF-1α, respectively. The selected dose of PDGF-BB used in the following experiments was chosen based on our previous FB/HA hydrogel release study [16], since the factor in the ex vivo osteochondral model was expected to be released over several days; while the SDF-1α concentration was chosen based on our previous study demonstrating that the factor could enhance MSCs migration in the intervertebral disc [39].

### 2.3. Ex Vivo Osteochondral Defect Model for Endogenous Cell Recruitment under Mechanical Loading

For ex vivo explant culture, 50 µL of FB/HA or FB/HA carrying chemotactic factors were cast into the osteochondral explants after defect creation. Then, osteochondral explant constructs were cultured in 3 mL of chondro-permissive medium and loaded in our bioreactor system. Osteochondral plugs underwent mechanical stimulation using a four-station bioreactor system, installed in a CO_2_ incubator at 37 °C, 5% CO_2_, 85% humidity [40]. A ceramic hip ball (32 mm in diameter) was pressed onto the osteochondral plugs to provide a constant displacement of 0.4 mm or 10% to 14% of the cartilage height (~3 to 4 mm), to fully maintain the contact of the ball with the hydrogel and the surrounding cartilage. Loading groups were exposed to axial compression in a sinusoidal manner between 0.4 mm and 0.55 mm, resulting in an actual strain amplitude of 10–13.7% or 14–18.3% of the cartilage height at a frequency of 0.5 Hz and concurrent shear motion by ball oscillation at ±25° and 0.5 Hz.

**Figure 1:**
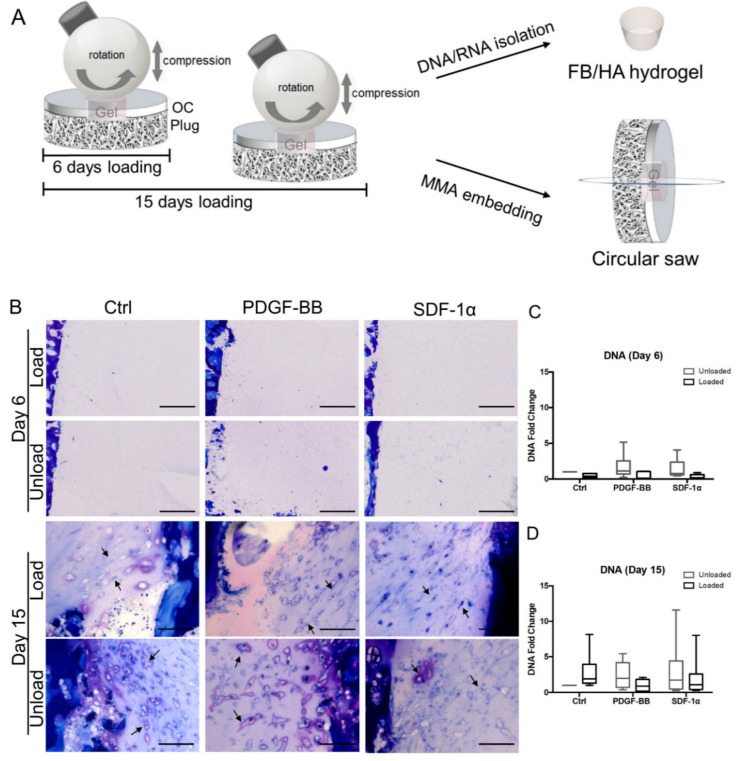
Effect of mechanical stimuli on cells migrating into an ex vivo osteochondral defect filled with FB/HA hydrogels. (**A**) Schematic representation of the experimental design used for cell migration experiments; OC Plug: Osteochondral explant; Gel: FB/HA hydrogel. (**B**) Representative images of osteochondral constructs stained with Toluidine blue (purple = glycosaminoglycan) showing cells infiltrating the defect after 6 and 15 days in presence or absence of mechanical stimuli; 20X magnification; scale bar indicates 100 µm. (**C**,**D**) Relative DNA content of unloaded and loaded cell infiltrating FB/HA hydrogels casted in the osteochondral defect models cultured for 6 and 15 days. Data were normalized to the DNA content of unloaded samples without chemokine addition. Results of 6 donors (day 6) and 10 donors (day 15) (one osteochondral explant per donor) are shown.

One hour of mechanical loading was performed per day over either 6 days or 15 days from the start of the culture (experimental scheme is represented in Figure 1A). In between loading cycles, samples were kept in free-swelling condition (no contact with ceramic ball). Unloaded explants with hydrogel served as controls. After loading, osteochondral explants were collected for DNA and RNA isolation or histological analysis.

### 2.4. Histology

Samples for histology were fixed in 4% buffered formaldehyde (Formafix AG, Hittnau, Switzerland) for 24 h, dehydrated until absolute ethanol, then embedded in methyl methacrylate (MMA) and sectioned in 130 µm sections. For staining, slides were treated with 1% formic acid and subsequently rinsed in tap water and dH_2_O. Toluidine blue staining was performed to visualize migrated cells and cartilage matrix. Briefly, slides were stained with 1% Toluidine blue for 1 min while heated at 55–60 °C on hot plate, rinsed in deionized water for 1 min and blot dried. Images were acquired using an optical microscope (Olympus, Tokyo, Japan).

**Figure 2:**
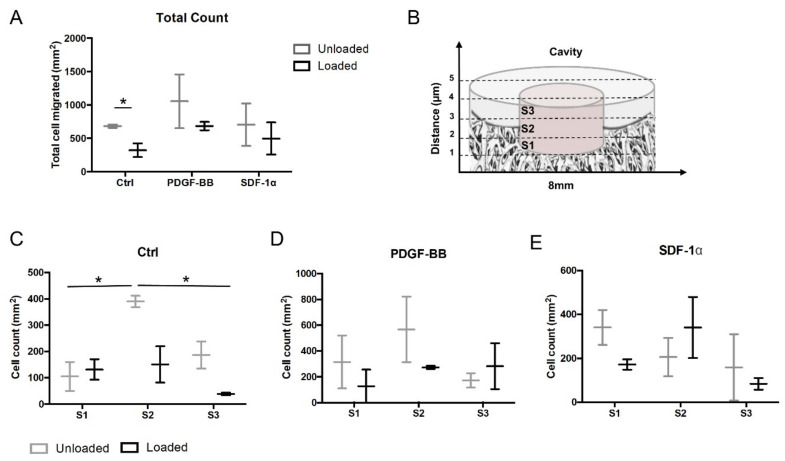
Cell colonization along the osteochondral defect depth. (**A**) Total count of cells invading FB/HA hydrogels into the osteochondral defect explants at day 15 of culture; * *p* < 0.05. (**B**) Schematic representation of the three different depth areas of the defect, S1, S2 and S3. (**C**–**E**) Cell count on histological sections alongside the bone layer S1, intermediate layer S2 and the cartilage layer S3 in the osteochondral defects at 15 days of culture in presence or absence of PDGF-BB or SDF-1α; * *p* < 0.05. Results from three donors (one explant per donor) are shown.

The number of the infiltrated cells was determined using Fiji software (National Institutes of Health, Bethesda, MA, USA). Cell colonization into the defect was assessed at day 15 by counting cell infiltration number in the defect area following specific criteria. Osteochondral defects of Toluidine blue stained cross-sections (*n* = 3/group) were divided in three subsections of 1 mm height (S1, S2 and S3; Figure 2A). The number of migrated cells per explant was defined as the sum of the numbers of migrated cells in three sagittal sections of the explant. RGB images were converted in 8-bit by using a trainable Weka segmentation plugin, in order to extract results by excluding the background (Toluidine blue staining) and selecting the area of interest (in this case the cells), as previously described [16].

### 2.5. RNA Extraction and Gene Expression Analysis

After 15 days of culture, FB/HA hydrogels were removed from the explant, homogenized using the Tissue Lyser system (Qiagen, Retsch, Hilden, Germany), and total RNA of the migrated cells was extracted using AllPrep DNA/RNA Micro Kit (Qiagen). RNA concentration and quality were measured using NanoDrop 1000 spectrophotometer (ThermoFisher, Waltham, MA, USA). cDNA was prepared using SuperScript Vilo IV Master Mix (ThermoFisher) according to the manufacturer’s instructions and real time PCR was performed on a Quant Studio Flex 6 instrument (ThermoFisher). Table 1 shows the sequences of bovine primers and TaqMan^®^ probes for collagens type-I (*COL1A2*), type-II (*COL2A1*), aggrecan (*ACAN*), matrix metalloproteinase 3 (*MMP-3*), *MMP-13,* and the catalogue numbers of the gene expression assays used for amplification of ribosomal protein lateral stalk subunit P0 (*RPLP0*), versican (*VCAN*), *β*1-integrin (*TFB1M*), and beta-actin (*ACTB*) (Applied Biosystems, Rotkreuz, Switzerland). Data collected at day 15 were expressed as relative values of target mRNA and determined according to the comparative C_T_ method. First the target gene expression was normalized to the expression of the reference gene *RPLP0*. This reference gene had been shown to remain stable under mechanical loading conditions, whereas other commonly used reference genes such as glyceraldehyde 3-phosphate dehydrogenase *GAPDH* may be affected by mechanical load [41]. In a second step the normalized target gene expression levels of samples treated by load and/or chemokine were expressed relative to the corresponding control sample for each donor. The control sample was neither treated by load nor by chemokine delivery. In this way, inter-donor variation was excluded, while only the effect of load and/or chemokine was assessed.

### 2.6. DNA Content Measurement

Hydrogels were assessed for DNA content after removing the FB/HA hydrogel from the osteochondral explants followed by homogenization in a Tissue Lyzer for sample disruption (Qiagen, Retsch, Hilden, Germany). DNA was purified using AllPrep DNA/RNA Micro Kit (Qiagen), and its content measured by Qubit 1X dsDNA HS assay kit following manufacturer’s instruction (Qubit 4.0 Fluorometer, ThermoFisher).

### 2.7. Statistical Analysis

Data were analyzed by using SPSS software, and the results are expressed as mean ± standard deviation (SD). Two independent experiments were performed using triplicates per group for early cell migration studies at day 6 and 15. Due to the non-symmetrical data distribution, a non-parametric test was selected to analyze the DNA content and the gene expression data. DNA amounts of samples treated with chemoattractant or mechanical load were expressed relative to the DNA content of untreated control samples from the same bovine donor to normalize for donor variation in basal cell migration. Similarly, gene expression data of samples treated with chemoattractant or mechanical load were expressed relative to the levels of untreated control samples. Independent samples were then statistically assessed by Kruskal–Wallis test and pairwise comparisons. For quantification of cells migrated into the osteochondral samples after 15 days of culture, three explants per group and three sections per sample were used; statistically significant differences between unloaded and loaded groups were determined by Kruskal–Wallis test and pairwise comparisons. Statistical significance was considered for *p* < 0.05.

## 3. Results

### 3.1. Mechanical Stimuli Affect Early Cell Migration in an Ex Vivo Osteochondral Culture Model

To determine the effect of loading on defect colonization and evaluate PDGF-BB and SDF-1α as efficient chemotactic factors for cells present in the ex vivo osteochondral explants, migrated cells were assessed as function of mechanical stress and chemoattractant delivery. To achieve that, osteochondral defect plugs filled with FB/HA hydrogel in presence or absence of 2 µg/mL PDGF-BB or 10 µg/mL SDF-1α were cultured for 6 and 15 days with or without exposure to mechanical stimuli. Toluidine blue staining revealed that endogenous cells interacted with FB/HA hydrogel; cells started adhering and infiltrating the defect within 15 days, while no or very few cells were visible at 6 days (Figure 1B). Mechanical loading seemed to influence the morphology of cells infiltrating the defect (day 15, Figure 1B).

To quantitatively assess the invasion of endogenous cells into the hydrogel delivered to the osteochondral defect explants, DNA measurement and cell counting were performed. DNA content analysis suggested that the exposure to mechanical stimuli tended to decrease cell recruitment at day 6 and day 15. Although a slight increase in DNA was found in the loaded compared to the unloaded control samples, these differences were not statistically significant (Figure 1C,D). The addition of chemotactic factors and their combination with mechanical stimuli did not show any effect on cell recruitment.

The cell colonization along the osteochondral explant depth at day 15 was further evaluated by histology. Sagittal sections of explants were cut to permit cell counting in order to explore endogenous cell migration in the entire defect (Figure 2A) and in three distinct depths of the defect (bone layer S1, interface layer S2 between calcified cartilage and bone, cartilage layer S3; Figure 2B). Total cell ingrowth was significantly lower in loaded control explants (without chemokine treatment) compared to unloaded controls (*p* < 0.05; Figure 2A). The numbers of migrating cells were generally more abundant at the interface layer between calcified cartilage and bone; indeed a significantly higher number of cells was observed in the unloaded control group (S2) when compared to unloaded controls in the S1 layer and to loaded control in the S3 layer (S1, S2, S3; *p* < 0.05, Figure 2C). In the adjacent layers (S1 and S3) of control constructs cell ingrowth was limited, and no significant differences were found. The addition of PDGF-BB into FB/HA hydrogel-constructs appeared to slightly increase cell infiltration in unloaded samples compared to the loaded plugs, albeit no significant differences were detected among the conditions tested (Figure 2A,D). The provision of SDF-1α had no effect on cell recruitment in unloaded samples, nor did it in loaded ones. Overall these results suggested that neither in presence nor in absence of applied stimuli, the chemotactic factors at the concentrations tested exerted any appreciable effects compared to control osteochondral constructs.

### 3.2. Biophysical and Biochemical Cues Influence Gene Expression within the Osteochondral Defect at Early Time Point

**Figure 3:**
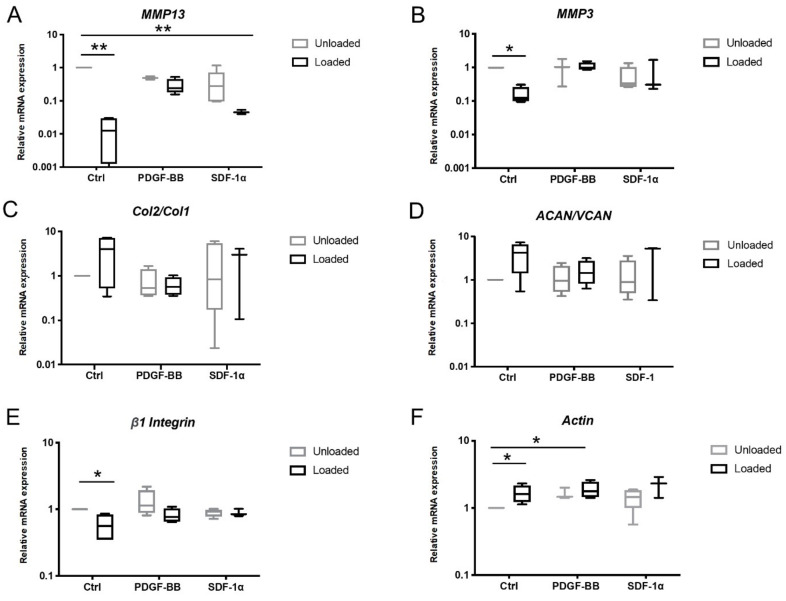
Effect of articular load and motion on phenotype of cells recruited into the wound site. (**A**–**F**) mRNA expression of cells infiltrating FB/HA hydrogels implanted into the osteochondral defect explant and exposed to complex load for 15 days. Data are expressed relative to mRNA levels of unloaded samples presented in the graphs by the first line. Results from four different donors (one osteochondral explant per donor) are shown; * *p* < 0.05, ** *p* < 0.01.

To test the phenotypic response of endogenous cells recruited in the FB/HA gel casted into osteochondral explants and uncover more closely the endogenous cartilage repair process, mRNA expression levels of the cells that migrated into the constructs were quantified after 15 days of loading (Figure 3). Ex vivo exposure of osteochondral explants to complex load led to strong decrease of catabolic markers in migrated cells by day 15. The effect was most evident for gene expression levels of *MMP13* in loaded constructs without or with SDF-1α compared to their respective unloaded samples (*p* < 0.01, Figure 3A); for mRNA levels of *MMP3*, only cells recruited in loaded control group showed significantly reduced expression (*p* < 0.05, Figure 3B). The mRNA ratios of *COL2A1* to *COL1A2* and *ACAN* to *VCAN* remained relatively stable (Figure 3C,D), whereas a significant reduction in β1 integrin and increase in actin expression were observed in loaded samples in absence or in presence of PDGF-BB compared to the unloaded control (*p* < 0.05, Figure 3E,F). These findings suggest that early applied mechanical stimuli altered the pro-adhesive phenotypic response of cells recruited into the osteochondral defect, thereby disfavoring the migration process.

## 4. Discussion

This study showed that complex articulating motion applied to an ex vivo osteochondral defect model, filled with hydrogel in presence or absence of chemoattractant, had a negative impact on endogenous cell recruitment into the wound site at early time point. In addition, the provided bioactive agents did not affect this process.

Our approach is based on the use of a previously described advanced platform [36], employed to monitor the spatiotemporal cell infiltration into the injury site under application of multiaxial compression and shear forces, using FB/HA hydrogels as matrix template and delivery carrier. It is widely accepted that mechanical loads are pivotal for cartilage regeneration; earlier described bioreactor studies focused on mechanical loading-based engineered tissue grafts for implantation in vivo or on mechanically stimulated osteochondral biopsy-related tissue maturation not combining complex motion patterns [42,43]. Others focused on a direct implantation of the osteochondral defect models in vivo [17,44,45]; to our knowledge there is no study intended to document ex vivo the influence of multiaxial stimuli on cell defect colonization. The advantage of using this pre-clinical tool is not merely to screen biomaterials and biomolecules, but also to closely study the dynamic process of cell homing, as in vivo experiments impede the ability to monitor cell migration and to detect the loads the tissues experience.

Histological analysis revealed that cells started migrating into the defect within two weeks of culture. Mechanical loading seemed to influence the morphology of the cells colonizing the defect; indeed, migrated cells in loaded samples assumed more spindle-shape morphology compared to samples which did not undergo loading and exhibited typical rounded and polygonal shape. Their different morphology suggests that mechanical input is one of the factors governing the mode of migration, in addition to cell type and hydrogel properties. Mesenchymal movement, used by spindle-shaped cells (such as fibroblasts) [46], appears to be dictated by the implementation of mechanical stimuli; whereas ameboid movement, both blebby and pseudopodal, which is used by elliptical-shape cells [12], may be more predominant in unloaded samples.

Quantitative DNA measurements did not show statistically significative differences at day 6 and day 15, even though fewer cells seemed to populate FB/HA hydrogels exposed to complex load. In support of our observations, total cell count indicated that complex articulating motion significantly decreased cell invasion in loaded control plugs compared to unloaded controls. These results suggested that mechanical stimuli negatively influenced cell migration by slowing down this process at early time points. We can, however, not exclude that this effect could be due to an inhibition of cell proliferation or an enhanced cell death [47].

It is important to mention that the SDF-1α and PDGF-BB gradients did not significatively enhance migration in our ex vivo model. Our previous in vivo study on osteochondral repair showed that the exposure of osteochondral defect explants with FB/HA hydrogels to 1 µg/mL PDGF-BB before implantation did not significantly enhance cell recruitment compared to untreated constructs [16]. Although that study used lower concentrations of PDGF-BB compared to the present study (2 µg/mL), the present findings are in line with our earlier observations indicating that cells colonize the defect without factor implementation and the tested factors do not improve cell recruitment. Interestingly, higher variations of numbers of migrating cells were noticed in the chemoattractant groups compared to the control groups without chemoattractant delivery (Figure 2). This may be attributable to different cellular responses to the chemotactic factors. The chemoattractant effect likely depends on the individual donor explant and on the presence of different proportions of cell types within the explants. In particular, stem and progenitor cells are known to be more responsive to chemotactic factors compared to mature cells [48].

The interface layer (S2) showed the highest cell invasion in unloaded control constructs in comparison to the adjacent layers, suggesting a new potential pattern of migration in the osteochondral unit where either cells present in the subchondral bone or in the calcified cartilage highly participate in defect restoration [49,50,51]. Previous models of cell recruitment in osteochondral defects mainly studied the migration of chondrocytes and subchondral bone derived cells, whereby the latter may include osteoblasts, osteoclasts, MSCs or even hematopoietic stem cells [52,53]. It is generally accepted that stem cells have the highest migration and proliferation rate, osteoblasts are assigned an intermediate rate, while chondrocytes undergo little migration or proliferation [53]. Nevertheless, certain growth factors have been shown to enhance chondrocyte migration [54]. Interestingly, fibrin sealant could promote migration of human chondrocytes in vitro, suggesting that the fibrin-based hydrogel supported the activity of the chondrocytes in our study [55]. Since the layers S1/S2 are mainly exposed to bone derived cells, while S2/S3 are affected by migrating chondrocytes, the interplay between the different cell types that includes autocrine and paracrine signaling may have promoted the cellular activity in the S2 area [51]. Nevertheless, although the migration of chondrocytes from pure cartilage explants is known to be slow, colonization of cells including progenitor-like cells could be demonstrated in a human cartilage explant model using a cell-free implant [56]. Finally, different cell migration rates may further be correlated with different timing of subchondral bone reconstitution and articular cartilage repair, which has been shown in an in vivo rabbit model of spontaneous osteochondral defect healing [57]. Future studies of osteochondral repair should focus on the origin of the reparative cells and mechanisms of cartilage and bone repair interactions over time. It is important to consider that subchondral bone and overlying hyaline cartilage are not two separate structures but a biological unit not only during embryogenesis, but also in adult life in support of the remodeling process.

The loading protocol was chosen based on previous protocols tested by our group. Antunes et al. investigated the effect of low intensity motion set-ups and a bioactive agent on gene expression of primary bovine chondrocytes seeded FB/HA hydrogels; due to the low resilience of the hydrogel, samples were subjected to an offset displacement of 10% and low amplitude dynamic axial compression between 10% and 11.5% [35]. Conversely, our previous work on the mechanically stimulated osteochondral explant culture model featured higher mechanical loading set-ups; due to mechanically stiffer polyurethane scaffolds and the use of osteochondral explants as more confined system, dynamic compression was applied at a strain amplitude between 10% to 20% or 14% to 26% [16]. We therefore tuned the mechanical loading protocol with applied complex motion of 10%-14% to fit the mechanical profile of the osteochondral defect constructs containing FB/HA hydrogel. In line with a previous study [58], the influence of external mechanical forces could dictate cell response by dampening matrix degrading collagenases involved in joint pathologies. It is worth noting that the effects of load on *MMP13* and *MMP3* gene expression were no longer present upon the addition of the chemokines. The joint motion simulator did not affect the mRNA ratio of Collagen II to Collagen I and Aggrecan to Versican, indicating minimal influence on the chondrocytic phenotype [59]. Our cell counting data indicated that cell infiltration was still low after 15 days, and due to their uneven distribution cells may not have been accessible to undergo strain-mediated chondrogenic differentiation and matrix remodeling.

Periodic mechanical stress may induce a reduction of endogenous cells adhesion in the defect at early time points by downregulating the expression of *β*1 integrin and upregulating actin expression. Since integrins provide the main molecular link attaching cells to extracellular matrix, and the bonds that link actin cytoskeleton to integrins dynamically break and reform [60], it is possible that extrinsic mechanical forces reduced integrin properties leading to altered mechano-sensing response as crucial determinants for cell migration.

Although any building blocks need physical forces in order to assemble and hold themselves together [61], we cannot exclude that the application of complex mechanical stimuli at early time point could trigger an altered biological outcome by physically breaking down early matrix organizational network that cells build up in favor of their migration. This is particularly enhanced in a hydrogel system set up, insufficient to counteract the imbalance of cell-generated tissue tension and dynamic load at high magnitude. Nonetheless, the mechanically stimulated osteochondral defect model mimics the entire joint only approximately, hence we cannot completely replicate the endogenous healing process as it happens in vivo. Cell migration is significantly influenced by the synovial microenvironment responsible for production of inflammatory cytokines and chemokines, which in turn trigger the cascade of events that could lead to invasion of endogenous reparative cells into the wound site [62].

Taken together these data suggest that the applied mechanical stimuli did not enhance cell recruitment into the osteochondral defect at early time point and the provided chemotactic agents did not influence this process. This might indicate that a well-orchestrated mechanical loading over time is crucial for successful design of endogenous cell recruitment and cartilage healing studies. After an observation period of 15 days, cell infiltration was evident, while the number of migrated cells was still limited. Due to this limitation, no extended evaluation of cell types, matrix synthetic activity and matrix composition could be performed besides the gene expression analysis. A parameter that needs more rigorous attention is the pre-culture time of the osteochondral hydrogel constructs, as it can play a pivotal role for tissue maturation and integration [63]. Indeed, a preliminary experiment showed that longer pre-culture of five weeks allowed more cell infiltration and matrix deposition into the injured site (Figure S1). To further observe this phenomenon, future studies will focus on the use of osteochondral explants filled with FB/HA hydrogel pre-cultured for longer time to assess load free effects before being subjected to mechanical stimuli.

## 5. Conclusions

The present short report details the temporal and spatial migration pattern in a mechanically stimulated ex vivo osteochondral defect explant filled with FB/HA hydrogel, demonstrating that loading post defect creation might inhibit the endogenous cell migration potential. The implementation of chemokines to increase cell migration was not efficient to overcome this negative effect. This study highlights a significant improvement in the understanding of osteochondral wound healing, suggesting that well-orchestrated mechanical application over time could be the prelude for enhancing cell mobilization and differentiation. The model is useful to decode the interplay between cells, hydrogel, mechanical and biochemical factors; it may unravel the dynamic process of endogenous cell recruitment and signaling pathways implicated in the repair. In light of the inherent advantages that could be utilized based on the modulation of different stimuli, the model represents an attractive system to improve our understanding about the management of joint injury and rehabilitation protocols. Longer-term studies will be required to assess hydrogel-guided neo-cartilage formation and neo tissue integration.

## Figures and Tables

**Table 1:** Oligonucleotide primers and probes used for qRT-PCR. COL: Collagen; ACAN: Aggrecan; MMP: Matrix metalloproteinase; VCAN: Versican; TFB1M: Beta-1-integrin; ACTB: Beta-actin. FAM: 6-carboxyfluorescein; TAMRA: 6-carboxytetramethylrhodamine.

Gene		Sequence or Cat. nr.
*COL1A2*	Primer forward (5′-3′)	TGC AGT AAC TTC GTG CCT AGC A
	Primer reverse (5′-3′)	CGC GTG GTC CTC TAT CTC CA
	Probe (5′FAM- 3′TAMRA)	CAT GCC AAT CCT TAC AAG AGG CAA CTG C
*COL2A1*	Primer forward (5′-3′)	AAG AAA CAC ATC TGG TTT GGA GAA A
	Primer reverse (5′- 3′)	TGG GAG CCA GGT TGT CAT C
	Probe (5′FAM- 3′TAMRA)	CAA CGG TGG CTT CCA CTT CAG CTA TGG
*ACAN*	Primer forward (5′-3′)	CCA ACG AAA CCT ATG ACG TGT ACT
	Primer reverse (5′- 3′)	GCA CTC GTT GGC TGC CTC
	Probe (5′FAM- 3′TAMRA)	ATG TTG CAT AGA AGA CCT CGC CCT CCA T
*MMP-3*	Primer forward (5′-3′)	GGC TGC AAG GGA CAA GGA A
	Primer reverse (5′-3′)	CAA ACT GTT TCG TAT CCT TTG CAA
	Probe (5′FAM- 3′TAMRA)	CAC CAT GGA GCT TGT TCA GCA ATA TCT AGA AAA C
*MMP-13*	Primer forward (5′-3′)	CCA TCT ACA CCT ACA CTG GCA AAA G
	Primer reverse (5′-3′)	GTC TGG CGT TTT GGG ATG TT
	Probe (5′FAM-3′TAMRA)	TCT CTC TAT GGT CCA GGA GAT GAA GAC CCC
*VCAN*	Cat. nr.	Bt03217632_m1
*TFB1M*	Cat. nr.	Bt03269747_m1
*ACTB*	Cat. nr.	Bt03279174_g1
*RPLP0*	Cat. nr.	Bt03218086_m1

## Supplementary Materials

The following are available online at https://www.mdpi.com/2073-4360/12/8/1754/s1, Figure S1: Osteochondral explant overview.
