# Supplementary material for: Mechanical Stress Inhibits Early Stages of Endogenous Cell Migration: A Pilot Study in an Ex Vivo Osteochondral Model
Source: Polymers (Basel). 2020 Aug 6;12(8):1754. doi: 10.3390/polym12081754 (PMC7466115; doi:10.3390/polym12081754)
Supplement: Supplementary file 1 [file polymers-12-01754-s001.pdf]

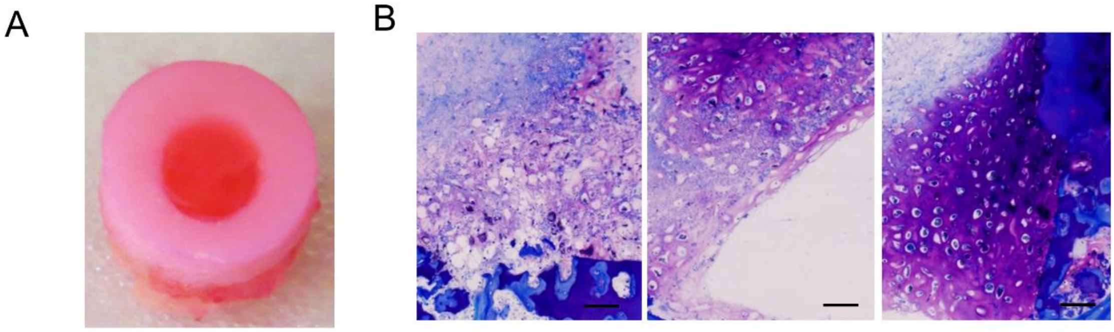

Supplementary Figure S1. Osteochondral explant overview. (A) Macroscopic images of osteochondral explant after 5 weeks of culture. FB/HA hydrogel becomes opaque in presence of cell invasion (left side). **(B)** Toluidine blue staining to assess endogenous cell migration after 5 weeks of culture. Cartilage formation in hydrogel is presented by pink/violet staining. 20X magnification, scale bar indicates 100μm.
